# Easy ultrastructural insight into the internal morphology of biological specimens by Atomic Force Microscopy

**DOI:** 10.1038/s41598-021-89633-2

**Published:** 2021-05-13

**Authors:** Fabian Christopher Herrmann

**Affiliations:** Institute of Pharmaceutical Biology and Phytochemistry, University of Münster, Münster, Germany

**Keywords:** Atomic force microscopy, Scanning probe microscopy

## Abstract

As a topographical technique, Atomic Force Microscopy (AFM) needs to establish direct interactions between a given sample and the measurement probe in order to create imaging information. The elucidation of internal features of organisms, tissues and cells by AFM has therefore been a challenging process in the past. To overcome this hindrance, simple and fast embedding, sectioning and dehydration techniques are presented, allowing the easy access to the internal morphology of virtually any organism, tissue or cell by AFM. The study at hand shows the applicability of the proposed protocol to exemplary biological samples, the resolution currently allowed by the approach as well as advantages and shortcomings compared to classical ultrastructural microscopic techniques like electron microscopy. The presented cheap, facile, fast and non-toxic experimental protocol might introduce AFM as a universal tool for the elucidation of internal ultrastructural detail of virtually any given organism, tissue or cell.

## Introduction

Our understanding of the detailed structural organization of biological material has ever since mainly been determined by microscopic techniques. Starting with light microscopy in the eighteenth century and culminating in the development of electron microscopes in the twentieth century, histology itself evolved alongside the improvement of microscopic techniques. Today, the plethora of our ultrastructural knowledge of organisms, tissues and cells is mostly derived from different electron microscopic approaches such as transmission or scanning electron microscopy.

Since the introduction of electron microscopy (EM), a large number of sample preparation techniques to gain suitable EM specimen have been established^1–5^. Nevertheless, the commonly applied protocols are still highly complex today, employing toxic chemicals and resulting in samples mainly suitable for electron microscopy, excluding other techniques and their correlation with EM data from the start. Additionally, ever so important immunolabeling investigations are hindered by several difficulties arising from the intense specimen treatment prior to EM data acquisition, especially from the embedding in epoxy resins^6, 7^. Altogether, EM suffers from certain critical limitations creating major obstacles concerning a variety of experiments on biological specimens.

With the introduction of Atomic Force Microscopy (AFM) by Binnig, Quate and Gerber in 1986^8^, a new microscopic approach became available incorporating certain striking advantages over classical ultrastructural techniques. With AFM, the application of vacuum to the sample in order to create imaging information was no longer needed, hence it finally became possible to gain ultrastructural data of biological material under physiological conditions^9, 10^. Additionally, the need for intense sample preparation techniques was substantially reduced compared to transmission electron or scanning electron microscopy (TEM, SEM). Further advantages of AFM over EM include e.g. the ability to precisely resolve z-axis data as well as the capability to access viscoelastic characteristics of a sample by force spectroscopy.

Besides the mentioned advantages of AFM over EM, one striking limitation still caused major problems in the acquisition of ultrastructural data of internal features of biological samples in the past. As a topographical technique, AFM needs to establish direct interactions between the measurement probe and the structure of interest in order to create imaging information. Gaining insight into the internal morphology of whole organisms, tissues and single cells by AFM has therefore been a common obstacle among AFM researchers.

Overcoming the mentioned problem promises abilities concerning the acquisition of ultrastructural data of biological samples by AFM compared to EM, e.g. the option to access precise z-axis data as well as the viscoelastic properties of a given sample only to name a few.

In order to enable AFM of internal features of biological material, a facile and broadly applicable polyethylene glycol embedding, ultra-sectioning and dehydration protocol was developed, resulting in the ability to ultrastructurally characterize the internal morphology of virtually any biological material by AFM. Initially, the specimens investigated in the study at hand were fixed using typical reagents like glutaraldehyde or paraformaldehyde. Secondly, sample dehydration was carried out by the application of a water/ethanol dilution series, followed by infusion of polyethylene glycol (PEG) as embedding medium. After block solidification and ultra-sectioning on a typical ultra-microtome equipped with standard glass knifes, the obtained sections were immobilized on coated glass slides and the water-soluble embedding medium was completely removed by repeated washing steps. Finally, the samples were dehydrated by a simple air flow technique and subsequently imaged by intermittent contact mode AFM under ambient conditions.

With the intention not to exclude scientists unable to access state-of-the-art AFM technology, commonly available AFM instrumentation (intermittent contact mode in air) was specifically employed in the presented investigations. The study at hand aims at clarifying the applicability of the mentioned protocol to selected representative biological specimens, in order to prove its feasibility on virtually any organism, tissue or cell.

## Results

### AFM-based ultrastructural imaging of the internal morphology of *Caenorhabditis elegans*

**Figure 1:**
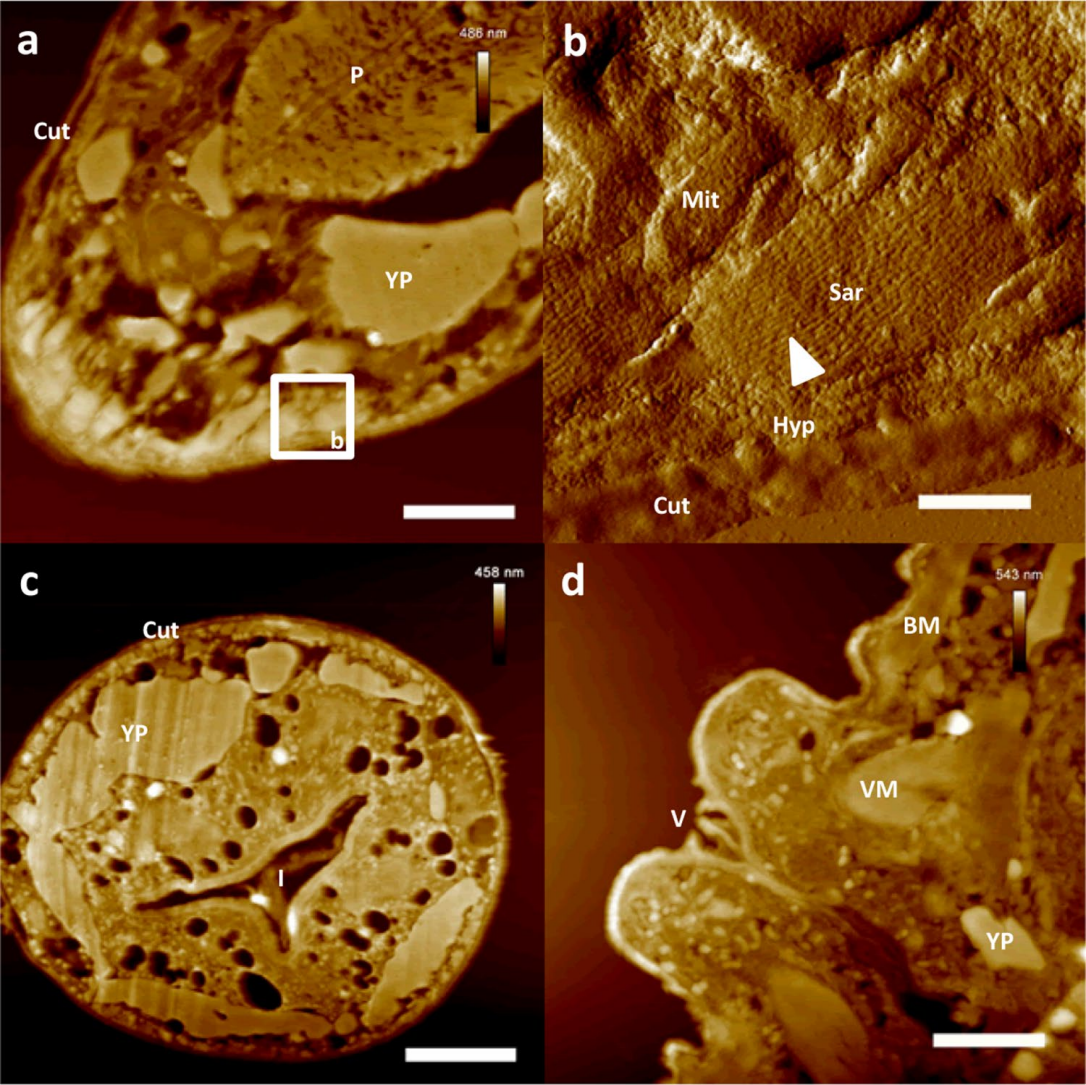
AFM-based ultrastructural analysis of *Caenorhabditis elegans* morphology. Nanoscopically resolved exemplary nematode tissues. (**a**) Height data of the head region of a *C. elegans* individual. (**b**) Close up on a single body muscle cell (amplitude data shown), with clearly resolved sarcomere morphology. Note the resolution of single myosin filaments (white triangle) within the sarcomere. (**c**) Height data of a cross section through the mid-region of a *C. elegans* individual. (**d**) Height data of a close up on the vulva of a *C. elegans* individual. Scale bars (**a**) and (**d**) 4 µm, (**b**) 600 nm and (**c**) 8 µm; *BM* body muscle, *Cut* cuticle, *I* intestinal lumen, *Mit* mitochondrium, *P* pharynx, *Sar* sarcomere, *V* vulva, *VM* vulval muscle, *YP* yolk pool, *white triangle* single myosin filament.

**Figure 2:**
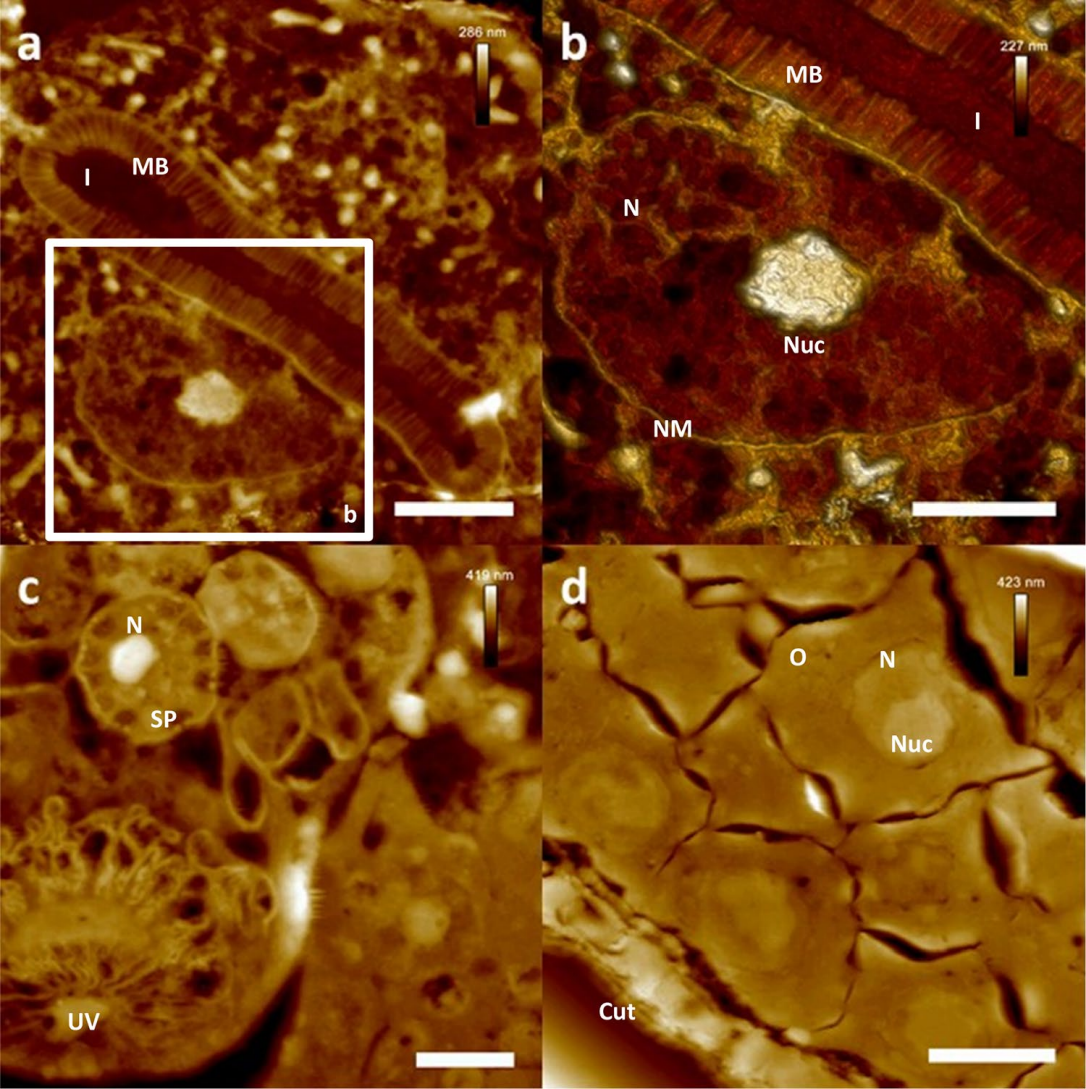
AFM-based ultrastructural analysis of *Caenorhabditis elegans* morphology. Nanoscopically resolved exemplary nematode tissues. (**a**) High resolution height data of two enterocytes forming the intestinal lumen. Note the clear resolution of single microvilli. (**b**) High resolution close up on nuclear region of a single enterocyte, showing ultrastructural details of the intranuclear composition. (**c**) High resolution height data of spermathecal tissue of *C. elegans*. Note the clear depiction of single spermatids (**c**). (**d**) Heigh resolution close up on gonadal tissue, showing single oocytes of the proximal gonads. Scale bars (**a**) and (**d**) 3 µm, (**b**) and (**c**) 2 µm. *Cut* cuticle, *I* intestinal lumen, *MB* microvilli brush, *N* nucleus, *NM* nuclear membrane, *Nuc* nucleolus, *O* oocyte, *SP* spermatid, *UV* uterine valve.

The ultra-sectioning, immobilization and dehydration of PEG-embedded *C. elegans* individuals yielded samples easily accessible under ambient conditions by tapping mode AFM, enabling the ultrastructural depiction of typical tissues of this nematode commonly used in life science experiments. The sample preparation allowed sectioning of a high amount of *C. elegans* individuals at once and resulted in a plethora of worm sectioning planes, permitting broad insight into internal features of the nematodes by light microscopy as well as by AFM. The morphological features investigated in detail by AFM showed ultrastructural information altogether in accordance with available TEM data of *C. elegans*^11–15^. Nanoscopic key features of the assessed tissues were clearly resolved by tapping mode AFM in air, e.g. single myosin filaments in sarcomeres of body wall muscle cells (Fig. 1b), the microvilli brush as well as details of the nuclear membrane and the intranuclear composition of enterocyte cells (Fig. 2a,b), single spermatids as well as the uterine valve (Fig. 2c) or the overall morphology of the cuticle (Fig. 1b). By the combination of phase contrast light microscopy with AFM, the identification of specific tissues in *C. elegans* ultra-sections was easily carried out, resulting in timesaving and precise AFM image acquisition. Due to this, specific organs and tissues like the gonads^12^, the vulva^16^, the pharynx^17^ or the intestine^11^ were easily identified and afterwards imaged nanoscopically by AFM (Figs. 1, 2).

### AFM-based ultrastructural imaging of exemplary mammalian tissues (human melanoma, human knee cartilage and mouse kidney)

To prove the applicability of the proposed technique towards mammalian organs and tissues, a human knee cartilage sample, a human melanoma biopsy and a mouse kidney were selected for further AFM-based ultrastructural elucidation. PEG-embedding as well as ultra-sectioning was easily carried out on the described mammalian samples, resulting in a high amount of AFM-suitable sections for further analysis.

**Figure 3:**
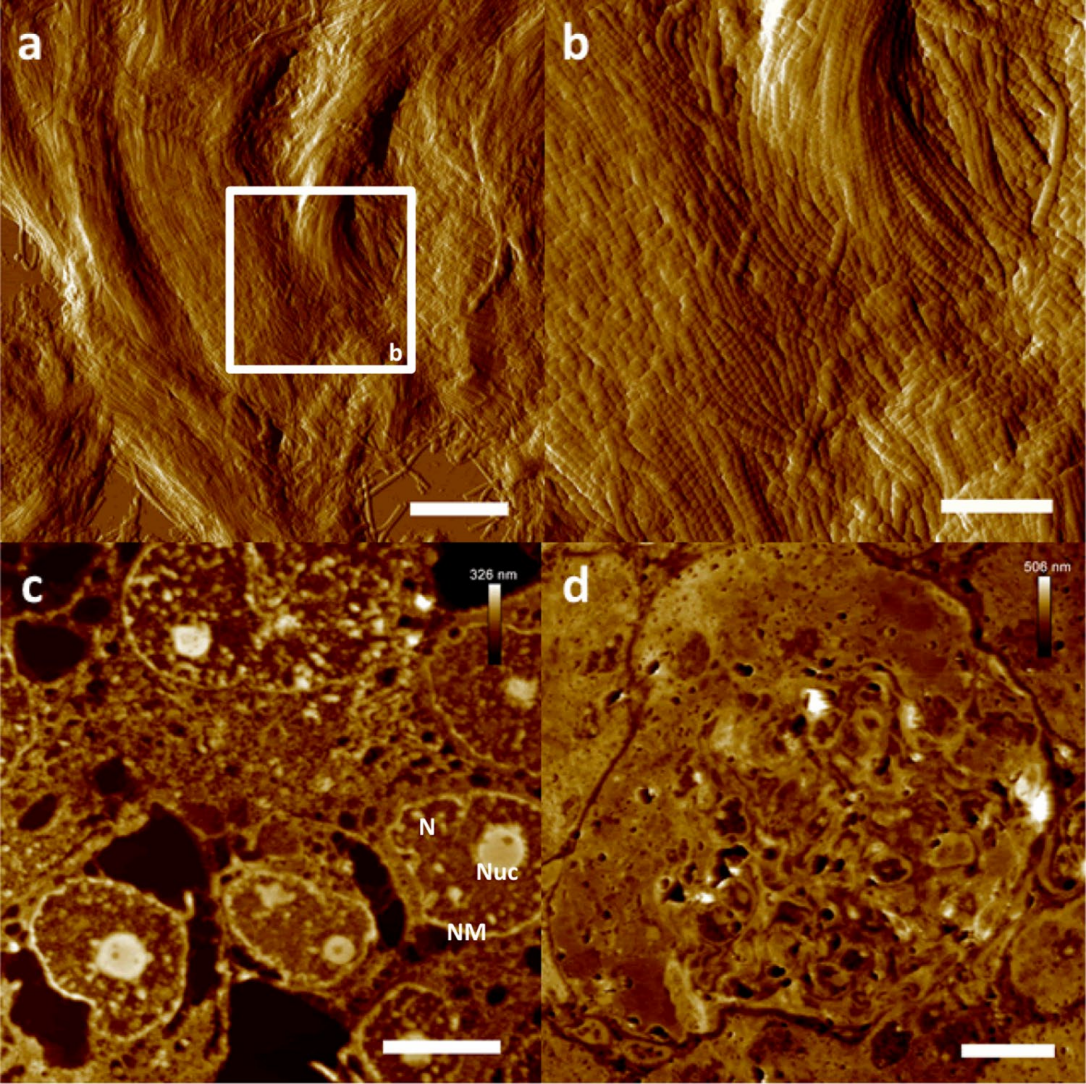
AFM-based ultrastructural analysis of exemplary mammalian tissues. Nanoscopic resolution on more complex tissues. (**a**) AFM amplitude data of an ultra-section of human knee cartilage. (**b**) High resolution close up on single collagen fibrils. Note the preservation as well as the clear resolution of the characteristic 67 nm D-periodicity of single collagen fibrils. (**c**) Height data depiction of an ultra-section from human melanoma tissue. (**d**) AFM height data of a mouse kidney ultra-section showing a single glomerulus as well as surrounding tubuli. Scale bars (**a**) 2 µm, (**b**) 840 nm, (**c**) 6 µm, (**d**) 10 µm. *N* nucleus, *NM* nuclear membrane, *Nuc* nucleolus.

In case of the cartilage sample, structural integrity of the collagen fibrils as well as of single collagen fibers was clearly preserved, showing not only the typical curled organization of collagen fibrils but also the expected ultrastructural detail (typical 67 nm D-periodicity) of single collagen fibers (Fig. 3a,b)^18, 19^.

The prepared human melanoma tissue was also nicely preserved after PEG-embedding and subsequent ultra-sectioning (Fig. 3c). The expected typical morphology of the nuclei (e.g. lobulated nuclear membranes) as well as the overall tissue composition additionally proved to be in accordance to the available literature^20–22^.

Ultra-sectioning of the PEG-embedded mouse kidney also yielded ultrastructural data proving the overall preserved tissue integrity (Fig. 3d). AFM image acquisition was again easily carried out in intermittent contact mode in air, showing glomerular ultrastructural morphology in accordance with TEM data from literature^23^.

### AFM-based ultrastructural imaging of the internal morphology of hedgehock ticks (*Ixodes hexagonus*, Ixodidae)

**Figure 4:**
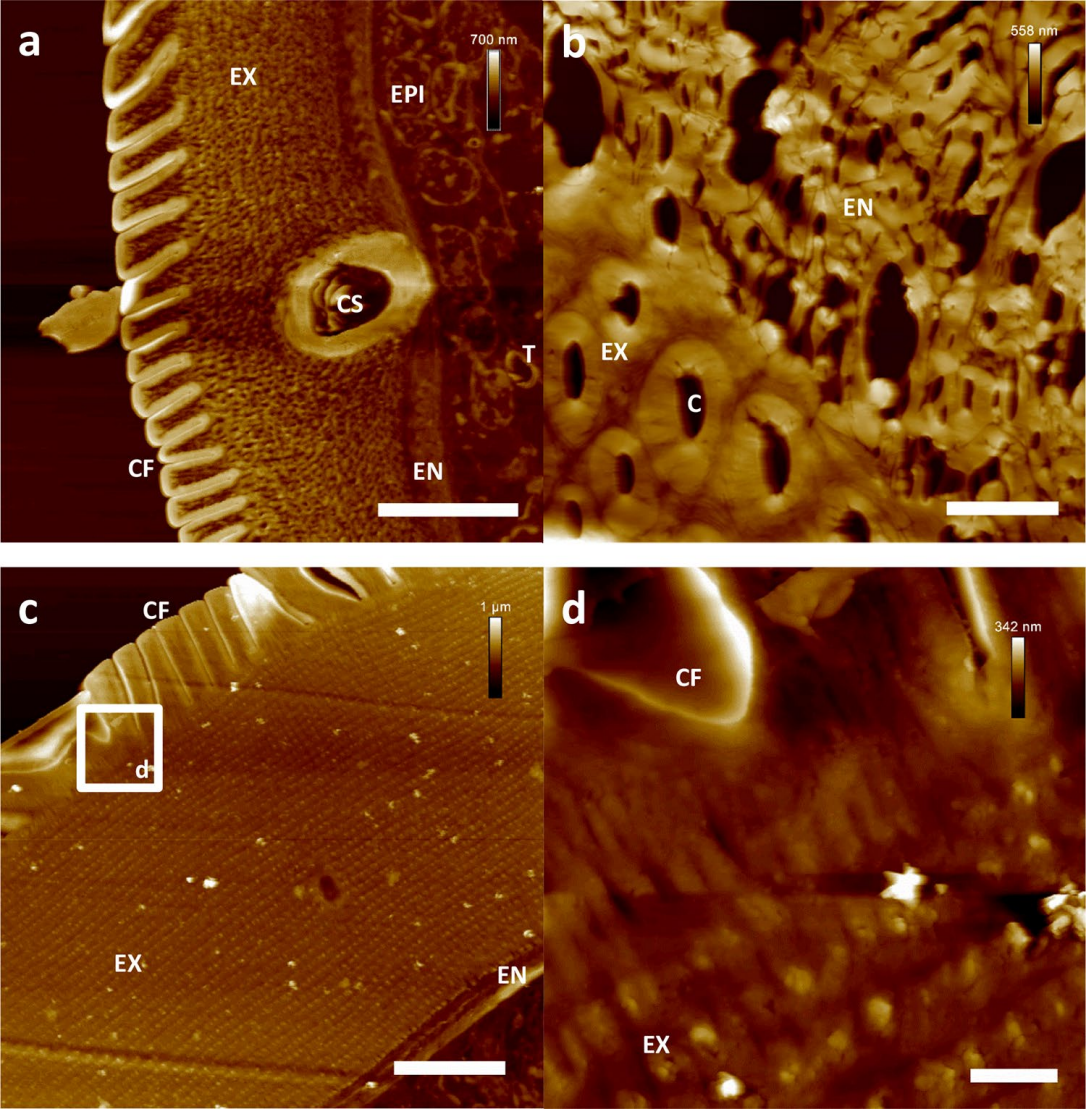
AFM-based ultrastructural analysis of the cuticle of hedgehog ticks (*Ixodes hexagonus*). Nanoscopic resolution on tissues from more complex organisms. (**a**) Cuticular detail of a starving nymph of *I. hexagonus* (**b**) Cuticular detail of a fully engorged adult individual of *I. hexagonus* (**c**) Cuticular detail of a starving adult individual (**d**) Higher resolution of the *I. hexagonus* cuticle. Scale bars (**a**–**c**) 10 µm, (**d**) 1 µm. *C* pore canal, *CF* cuticular folds, *CS* cuticular sensilla, *EN* endocuticle, *EPI* Epidermis, *EX* exocuticle, *T* single trachea.

In order to assess the possibilities and limitations of the applied ultra-sectioning protocol concerning larger and more complex organisms, hedgehock ticks (*Ixodes hexagonus*, Ixodidae) were selected for further ultrastructural investigations by AFM. In general, the overall morphology of the tick´s tissues is far more complex in comparison to the nematode *C. elegans*. In detail, especially differences in the hardness of tissues (e.g. of the cuticle compared to the inner organs) could have negatively affected the quality of the ultra-sections depending on the characteristics of the embedding medium. In case of the proposed PEG 4000 embedding protocol, the yielded ultra-sections of *Ixodes hexagonus* individuals showed nice preservation of tissue integrity, independent from hardness or composition. Ultrastructural elucidation by AFM resulted in the depiction of characteristic details of the tick’s cuticle as well as of internal organs. The cuticular folds, the exo- and endocuticle, the epidermis as well as single tracheae were nicely preserved in the investigated ultra-sections and could be observed in ultrastructural detail by AFM (Fig. 4). Altogether, the results of the AFM-based data acquisition were in accordance with available data from related TEM investigations on ticks of the genus *Ixodes*^24^.

### AFM-based ultrastructural imaging of the epidermal cell wall of *Senna alexandrina* (Fabaceae) leaves

**Figure 5:**
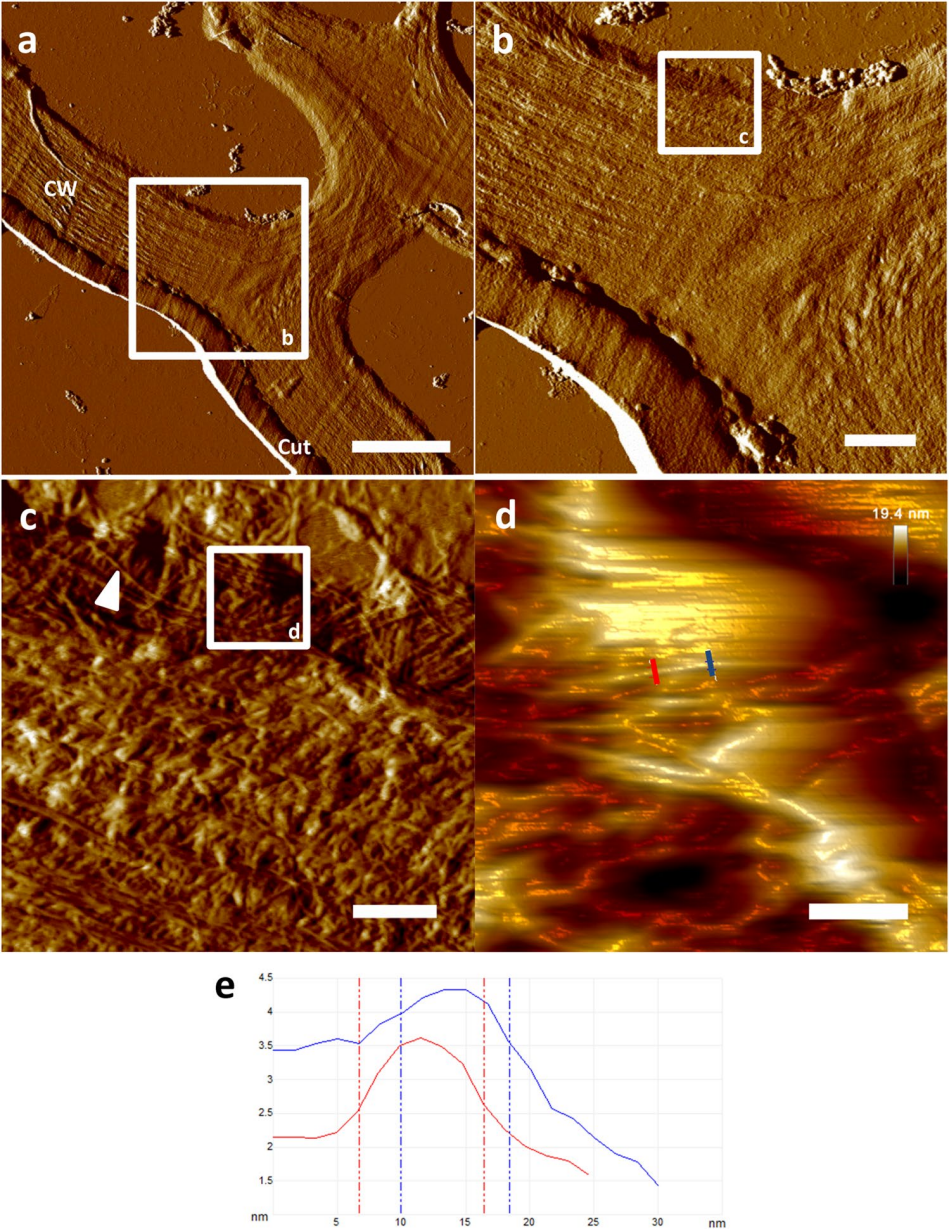
AFM-based ultrastructural analysis of the epidermal cell wall of *Senna alexandrina* (Fabaceae) leaves. AFM-data acquisition on cell wall ultra-sections allowed nanoscopic resolution down to the microfibril level. (**a**–**c**) Low, medium and high magnification amplitude data of *Senna alexandrina* cell wall morphology. Note the clear depiction of the layered cell wall composition and the resolution of single macro- as well as microfibrils (white triangle). (**d**) High resolution height data of single microfibrils (3D representation). (**e**) Section analysis of a single microfibril showing lateral dimensions of just below 10 nm. Note the corresponding z-axis resolution below 0.5 nm. Scale bars (**a**) 4 µm, (**b**) 1 µm, (**c**) 320 nm, (**d**) 100 nm. *Cut* cuticle, *CW* cell wall, *white triangle* isolated microfibril.

Plant tissues constitute another area of interest concerning the elucidation of ultrastructural morphology. In order to show the applicability of the protocol to tissues of plant origin, the epidermis of *Sennes alexandrina* (Fabaceae) was chosen for further investigation of the cell wall’s ultrastructure by AFM. Ultra-sectioning and dehydration of PEG-embedded leaves from *S. alexandrina* resulted in specimen showing nicely preserved cell wall morphology of the epidermis already by light microscopy. Subsequent AFM-based data acquisition depicted ultrastructural detail in accordance to morphological data known from the available literature^25–27^ (Fig. 5). Altogether, intermittent contact AFM of the prepared ultra-sections was able to resolve the fibril-composed structure of the investigated plant cell wall down to the nanometer level. Macro- as well as microfibrils were easily observed, allowing deeper insight into the overall ultrastructural composition of this histological detail of the *Senna alexandrina* epidermis (Fig. 5).

### AFM-based ultrastructural imaging of eu- and prokaryotic cells of representative cell culture experiments

To verify the potential use of the proposed protocol on the rather demanding level of single cells, exemplary pro- and eukaryotic cells were selected for further AFM-based ultrastructural investigations. As could be cautiously expected from the preceding experiments on complex organisms, the used PEG-embedding also allowed the ultra-section and subsequent AFM-based data acquisition of single cells. The resulting ultra-sections of *Euglena gracilis* and of *Escherichia coli* cells showed overall nice preservation of the cellular integrity as well as of intracellular details.

**Figure 6:**
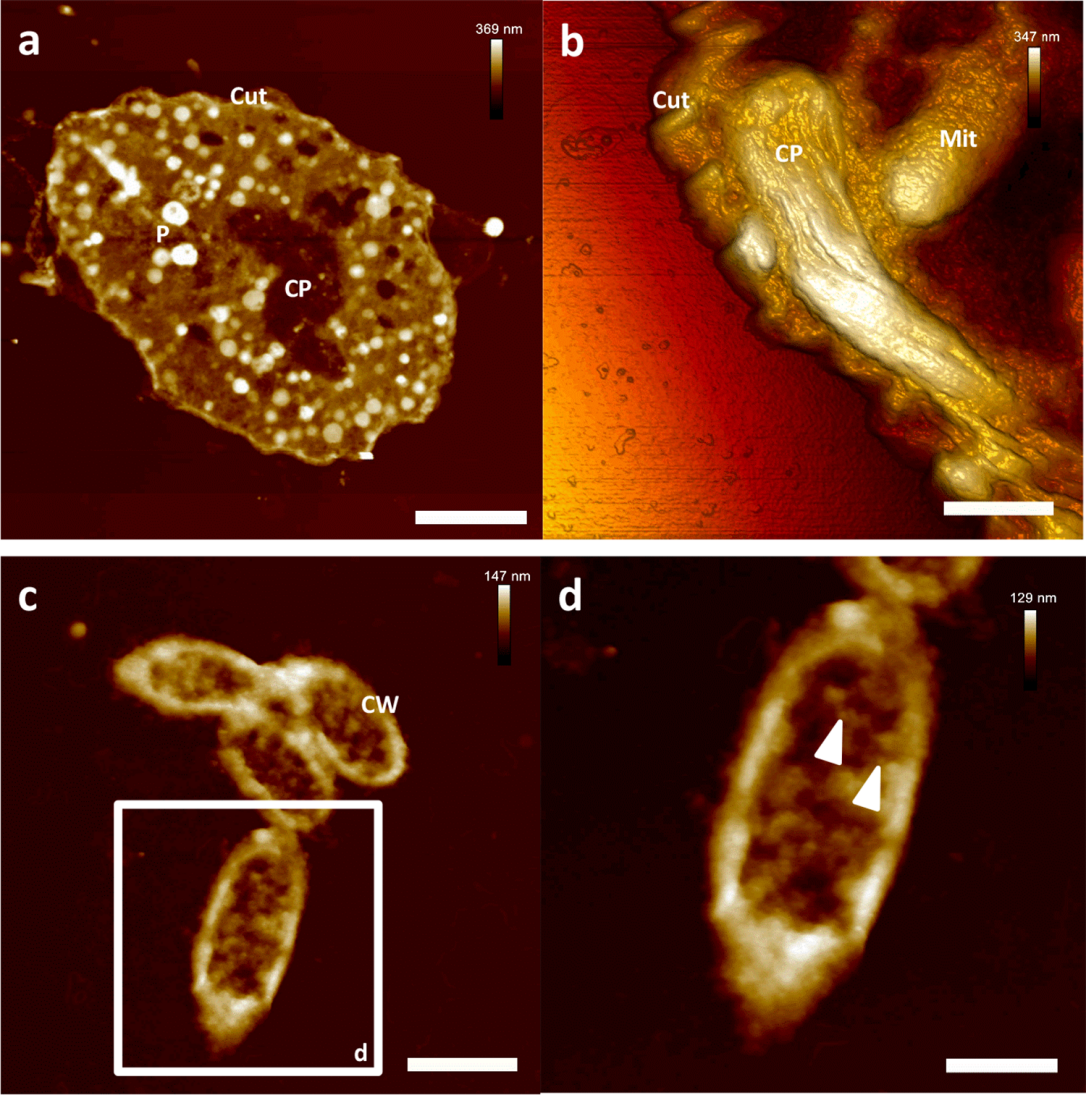
AFM-based ultrastructural imaging of eu- and prokaryotic cells of representative cell culture experiments. Nanoscopic resolution of the internal morphology of single cells. (**a**) Cross section of a single *Euglena gracilis* cell. (**b**) High resolution detail of an ultra-section from *E. gracilis* (3D representation). (**c**) Ultra-section of a group of *E. coli* bacteria. (**d**) Close up on a single *E. coli* individual. Note the depiction of single particles around 20 nm in diameter, most probably ribosomes (white triangles). Scale bars (**a**) 6 µm, (**b**) 940 nm, (**c**) 1 µm and (**d**) 520 nm. *CP* chloroplast, *Cut* cuticle, *CW* cell wall, *Mit* mitochondrium, *P* paramylum.

In case of the studied *E. gracilis* cells, ultrastructural details of the cuticle, the chloroplasts and of vacuoles and vesicles were easily accessible by AFM under ambient conditions. The observed intracellular morphology of the mentioned specimen was altogether in accordance with available TEM data of *E. gracilis*^28, 29^ (Fig. 6a,b).

The preparation of ultra-sections of single *E. coli* cells proved to be more complex compared to larger eukaryotic cells. In order to allow sufficient insight into this considerably smaller organisms, ultra-sections below 100 nm in thickness needed to be prepared. In case of the *E. coli* experiments, a thickness of roughly 70 nm was experimentally determined to be suitable for AFM-data acquisition on sectioned *E. coli* cells. AFM of the mentioned sections resulted in the depiction of typical morphological details expected from ultrastructural analysis of *E. coli* cells by TEM^30^. The cell wall as well as the general intracellular composition were overall in accordance to available low power TEM data^30^ (Fig. 6c,d).

## Discussion

In order to obtain fast, facile as well as cheap nanoscopic insight into internal features of organisms, tissues and single cells by atomic force microscopy, a combination of polyethylene glycol embedding, ultra-sectioning, immobilization, dehydration and subsequent data-acquisition in ambient conditions by intermittent contact mode AFM was successfully applied to a variety of representative biological specimens.

Sectioning techniques have already been commonly used for the preparation of AFM suitable samples from different origin in the past. In general, mainly protocols derived from electron microscopic approaches have been applied to create surfaces accessible by AFM, e.g. for nanoscopic analysis in the field of materials science^31^. Epoxy-based approaches have also been employed to study biological samples (e.g. *C. elegans*^32^) but suffered from specific limitations concerning embedding resins remaining in the analyzed sections, deeming the application of a topographical technique like AFM especially difficult. Cryo-sectioning techniques have also been applied in some studies to elucidate internal features of tissues by AFM^33, 34^. These techniques altogether suffered to a certain extend from being experimentally highly complex concerning the sample preparation as well as the needed expensive machinery e.g. for cryo-sectioning, probably resulting in the rather scarce application of this methodologies in the past. In contrast to that, although being a particularly suitable embedding medium e.g. due to its high solubility in water, PEG embedding protocols have only very rarely been used in order to create AFM suitable specimen in the past^35–37^. Apart from the publications by our institute^36^^,^^37^, only one article from the early days of AFM research seems to exist on PEG embedding combined with AFM data acquisition^35^. Besides different AFM machinery and measurement techniques employed in the mentioned study by Ushiki et al*.*, another and far more complex and challenging dehydration technique (critical point drying) has been applied. Altogether, PEG embedding has only scarcely been used in combination with AFM in the past.

As a result of the PEG technique applied in this study, immobilized and embedment-free sections were yielded, thereby generating close to optimal measurement characteristics concerning subsequent data acquisition by AFM. As one of the main advantages of the described protocol, this resulted in the ability to gain high quality ultrastructural AFM-data of internal characteristics of organisms, tissues and single cells. Another advantage of the AFM-based data acquisition on embedment-free ultra-sections was the accurate depiction of z-axis data nearly inaccessible by electron microscopic approaches. The overall resolution in air so far reached by the approach can be specified from the presented results of this study to be laterally at least below 10 nm and vertically at least below 0.5 nm (Fig. 5d,e). Future improvements concerning the achievable resolution could be realized by the use of cantilevers with smaller curvatures as well as by the application of more sophisticated AFM measurement techniques in liquid (e.g. PeakForce Tapping).

One key consideration to evaluate preparations for subsequent (ultra)structural assessment by any microscopic technique is the possible introduction of artifacts by sample pretreatment. More detailed, fixation, embedding, sectioning and dehydration procedures need to be taken into account as possible sources of morphological changes to the sample. In case of the proposed technique, common and validated fixation techniques (e.g. paraformaldehyde or glutaraldehyde) have been employed and can therefore be ruled out as specific sources of new artifacts^38, 39^. The applied PEG-embedding itself may also be responsible for the introduction of artifacts. However, compared to TEM epoxy preparations, the proposed PEG-embedding technique does not require the use of toxic or tissue incompatible solvents and is therefore in general less prone to artifact introduction. Additionally, PEG itself is characterized by specifically advantageous properties such as a particularly high tissue compatibility as well as the low tendency to shrinkage during solidification^40–42^. Sectioning on ultra-microtomes employing typical glass knifes is also known to introduce typical artifacts in the course of ultrastructural image acquisition. In case of the presented technique, the common “streaks” in sample preparations resulting from small chips broken from the knife edge were also sometimes detected (e.g. Figs. 1c,  4c). Altogether, this artifact can be normally clearly recognized as a result of the employed glass knifes e.g. due to parallel streaks in the image data. Additionally, this artifact seemed to be rather rare compared to typical epoxy preparations, which may be due to the rather low hardness of the embedding material PEG 4000. Finally, dehydration techniques can be relevant sources of tissue damages and artifacts. In case of the technique applied in this study, dehydration of the ultra-sections was rapidly performed by simple evaporation under continuous pressurized airflow. In case of the air-based dehydration of the immobilized ultra-sections of this study, the artifacts introduced by this technique seemed to be negligible when comparing the AFM results to available TEM data e.g. of *C. elegans*^11–17^ as well as to results obtained in our lab employing an alternative and validated but far more complex dehydration technique (hexamethyldisilazane (HMDS) dehydration^36, 43^, data not shown). The main reason for this might be given in the very small thickness of the prepared ultra-sections and their resulting tendency to dry rapidly. It is known from the development of other dehydration techniques, e.g. critical point drying, that the slow evaporation of liquids (specifically due to the surface tension occurring during the transition from liquid to gaseous state) introduces high local forces on the specimen, therefore being one typical source of common dehydration artifacts^43^. In case of the ultra-sections of this study (thickness down to 70 nm), dehydration by continuous airflow resulted in ultrafast drying, possibly limiting the negative evaporation effects to a minimum. The facile, fast and most of all cheap and unchallenging airflow technique employed in this study can therefore be considered to be at least a suitable alternative for the dehydration of ultra-sections of PEG-embedded specimens for subsequent nanoscopical analysis by AFM. Altogether, the results at hand indicate a rather low susceptibility of the technique to artifact introduction.

Compared to the more complex and invasive sample preparations commonly used for TEM investigations, the protocol applied in this study incorporated some specific advantages besides gaining AFM-suitable preparations. More detailed, the applied technique uses only non-toxic, broadly available and most of all extremely cheap chemicals (specifically polyethylene glycol 4000 and ethanol). Besides being non-toxic and highly economical, the ability of the methodology to gain internal ultrastructural data of biological specimen in only about 4 h from fixation to AFM data acquisition should also be mentioned. In contrast to typical TEM preparations, ultrastructural insights are therefore easily possible within a single day. Additionally, the overall work load of the proposed AFM preparation is at least to some extend reduced compared to standard epoxy embedding techniques e.g. by the missing need for post-fixation treatment. Furthermore, specific preparation of the embedment material is not necessary. While typical epoxy resins have to be prepared before use (e.g. by mixing resin and polymerization agents), PEG can be applied directly to the specimens after melting or solvation in H_2_O. Altogether, the proposed technique incorporated certain advantages compared to classical TEM epoxy embedding procedures such as the overall reduced costs and toxicity, as well as the reduced work load and the needed preparation time.

As a further general advantage of the PEG methodology, the possibility to directly interact with the sample´s morphology exposed by ultra-sectioning and subsequent removal of the embedding medium might enable additional experimental approaches not commonly available so far. This could facilitate the chemical characterization of exposed intracellular details e.g. by AFM-IR or Raman techniques, sophisticated intracellular assessments like MicroRNA quantification as well as the correlation of this data with ultrastructural AFM detail in the future.

Another relevant limitation given with common TEM approaches and resulting from the invasive sample preparation is the difficulty to perform localized immunolabeled protein detection on epoxy-embedded specimens. In contrast to that TEM-associated limitation, the proposed AFM-based technique should allow the facile correlation of immunohistochemistry with subsequent ultrastructural AFM investigations. PEG-embedding protocols have proven to preserve the antigenicity of prepared tissues to a high extend, therefore enabling the detection of specifically localized antigenicity e.g. by epifluorescence microscopy^41^. In case of the commonly realized instrumental correlation of an AFM with an inverse optical epifluorescence microscope, antigen staining, subsequent identification by epifluorescence microscopy and finally co-localized ultrastructural data acquisition by AFM should become easily possible. This could allow the direct correlation of intracellular protein localization and corresponding high-resolution ultra-structural AFM-detail in the future.

Another advantage of the proposed protocol was given in the rather undemanding ultra-sectioning of the PEG-embedded specimens. In general, ultra-microtomy is a quite complex and challenging technique. The production, handling and especially the transfer of ultra-sections to e.g. glass slides or TEM grids can be particularly tedious^44^. In case of the presented PEG-protocol, the ultra-sectioning proved to be quite simple, allowing the creation of long sectioning bands, which were easily transferrable to a prepared substrate. No orientation or further handling of single sections was needed, allowing the technique to be applied even by unexperienced users after only a short time. Additionally, the proposed PEG-embedding enabled a wide variety of section thicknesses in a reproducible manner, deeming the technique suitable for samples of various origins.

Besides the mentioned positive aspects, some disadvantages of the protocol also need to be taken into account. One limitation of the technique is the at least partial inaccessibility of the AFM-suitable preparations to TEM experiments. This is mainly given by the fact, that post-fixation steps prior to embedding procedures are typically needed for sufficient image contrast in TEM investigations (uranyl acetate and/or osmium tetroxide). In case of the proposed protocol, these invasive preparation steps have been specifically left out in order to reduce the work load as well as the toxicity of the preparation protocol but also because of potential changes introduced to the sample by this invasive chemicals potentially leading to problems with AFM image acquisition as well as with future correlative experiments like immunolabeling. Direct correlations between AFM and TEM data of a single sample preparation are therefore generally restricted, being the reason for the missing direct AFM and TEM comparisons in this study. On the other hand, immobilized and dehydrated ultra-sections should be suitable for SEM analysis after sputter coating. Another disadvantage of the presented methodology is the tendency of ultra-sections to overlap when being immobilized on coated glass slides, sometimes leading to difficulties in the identification of specific tissues in a single ultra-section.

In summary, the presented protocol allowed specific AFM-based ultrastructural insight into internal features of whole organisms as well as tissues and single cells. The achieved lateral resolution was specified to be at least below 10 nm accompanied by a vertical resolution of below 0.5 nm, possibly allowing further resolution improvements by the application of more specialized AFM measurement techniques in the future. One main advantage of the proposed technique, besides the fast and facile ultrastructural insight into a variety of biological specimens by AFM, was the possibility to correlate light microscopic experimental options (e.g. epifluorescence microscopy) with co-localized ultrastructural image acquisition. Altogether, the investigated approach constitutes a facile, fast and cheap alternative to classical TEM assessments of internal ultrastructural morphology of complex organisms, tissues as well as single cells and might enable interesting new experimental options for the analysis of intracellular details in the future.

## Methods

### Chemicals

If not stated otherwise, all chemicals were purchased from Merck KGaA (Darmstadt, Germany).

### Fixation

#### Caenorhabditis elegans

Fixation of *C. elegans* individuals was carried out after removal of the M9 growth medium (washing three times with Aqua Millipore) via application of glutaraldehyde solution (4% in PBS) for 10 min under gentle agitation at room temperature.

#### Mammal tissue samples (human knee cartilage, human melanoma, mouse kidney)

Fixation of the presented mammal samples was carried out in freshly prepared paraformaldehyde solution (4% in PBS, pH 6.9, paraformaldehyde for synthesis) for 60 min under gentle agitation at room temperature.

#### Ixodes hexagonus

The tick samples of this study were fixed by the application of freshly prepared paraformaldehyde solution (4% in PBS, pH 6.9) for 60 min under gentle agitation at room temperature.

#### Sennes alexandrina

Fixation of *S. alexandrina* leaves was carried out using a freshly prepared mixture of 90 mL ethanol (70%, absolute EtOH pro analysi), 5 mL formaldehyde (36%) and 5 mL glacial acetic acid (H_2_O-free, pro analysi) for 24 h under gentle agitation at room temperature.

#### Euglena gracilis and Escherichia coli

*Euglena gracilis* and *E. coli* cells were fixed after the removal of growth medium (washing three times with Aqua Millipore) by applying freshly prepared paraformaldehyde solution (4% in PBS, pH 6.9) for 10 min under gentle agitation at room temperature.

### Polyethylene glycol embedding

Polyethylene glycol (polyethylene glycol 4000 for synthesis) embedding was executed employing absolute ethanol as intermedium. Prior to the embedding procedure, each sample was washed at least twice with Aqua Millipore followed by gentle agitation for at least 15 min to remove the remaining fixative completely. Subsequently, an ethanol dilution series was applied (absolute EtOH pro analysi, EtOH/H_2_O 15:85, 25:75, 50:50, 70:30, 95:5 and 100% EtOH, 15 min for each dilution applied under gentle agitation). In case of the small samples (e.g. *C. elegans*), change of dilutions was performed by centrifugation (3000×*g* for 5 min), subsequent removal and disposal of the supernatant followed by the addition of the next dilution step. PEG infusion was finally introduced by the application of two PEG/EtOH (50:50) steps followed by altogether two final steps of absolute PEG 4000 (polyethylene glycol 4000 for synthesis). The PEG containing steps were infunded at a constant temperature of 64 °C for 15 min under gentle agitation. During the preparation of *C. elegans* or single cells, changing of the PEG-containing solutions was carried out via centrifugations in a -rotor preheated to 64 °C (Eppendorf MiniSpinPlus with Rotor F-45-12-11, Eppendorf AG, Hamburg, Germany). The resulting samples suspended in absolute and molten PEG 4000 were finally transferred into a capsule of choice for solidification at room temperature [either BEEM Embedding Capsules Size 00 (Electron Microscopy Sciences, Hatfield, PA, USA) or 1.5 mL conical reagent tubes (Eppendorf AG, Hamburg, Germany)]. To prepare small samples, a final centrifugation at 3000×*g* in a rotor preheated to 64 °C was carried out in order to ensure the orientation of the specimens in the apex of the capsule before ultra-sectioning. In contrast to that, larger samples (e.g. mammalian tissues) were easily positioned using preheated tweezers instead of a more complex centrifugation procedure. After solidification at room temperature, the embedding blocks were removed from the capsules and a small aluminum rod matching the block’s diameter was melted onto the basis of each block in order to allow mounting in the specimen holder of the ultra-microtome used for subsequent sectioning.

### Ultra-sectioning

Serial ultra-sectioning of the PEG-embedded specimen was performed with a Reichert/Leica Ultracut E microtome (Leica Microsystems, Wetzlar, Germany) equipped with typical glass knifes (glass strips 7890-04, LKB Bromma, Stockholm, Sweden), freshly prepared on a LKB Bromma 7801B KnifeMaker (LKB Bromma, Stockholm, Sweden). An inclination angle of 2–3 degrees and a section velocity of around 50 mm/s was used to gain serial sections ranging from 70 to about 750 nm in thickness, depending on the sample of interest. All sectioning operations have been carried out under ambient conditions resulting in easily and reproducibly obtainable ultra-sections.

### Dehydration and immobilization of the ultra-sections

A section band including around 200 sections was afterwards transferred into a 40 µL droplet of Aqua Millipore on a poly-l-lysine coated glass slide (Polysine slides, Gerhard Menzel GmbH, Braunschweig, Germany) using a human eye lash glued to a wooden stick. After the transfer of the section band into the droplet, a coverslip (24 × 40 mm) was gently applied and left on the sample for at least 5 min. This resulted in the adhesion of the ultra-sections to the coated glass slide. The cover slip was subsequently gently removed by the immersion of the whole sample in Aqua Millipore, leading to the flotation of the cover slip which was afterwards easily removed using a pair of tweezers. The resulting sample can be used at this point for further staining methodologies before dehydration. In case of the study at hand, the samples were afterwards dehydrated by evaporation using mildly pressurized air flow (hand bellows).

### AFM data acquisition

AFM image acquisition of the dehydrated ultra-sections was performed in intermittent contact mode (Tapping mode) under ambient conditions employing a Veeco/Bruker Bioscope equipped with a Nanoscope IIIa controller and n-type silicon cantilevers (HQ:NSC14 Al BS, nominal tip radius below 10 nm, nominal spring constant 5 N/m, resonance frequency around 160 kHz; µmesh, Sofia, Bulgaria) coupled with an inverted fluorescence microscope (Zeiss Axiovert 135, Carl Zeiss Microscopy GmbH, Jena, Germany). AFM image acquisition was carried at an oscillation 2% below the cantilever´s resonance frequency, using a free RMS amplitude of roughly 2 V, scanning rates around 0.5 Hz and amplitude setpoints around 1.2 V. All measurements were conducted in a laboratory ranging from 40 to 60% in humidity.

### Processing of AFM images

Processing of AFM imaging data (plane fit, flattening and crop operations) was carried out employing the software Nanoscope Analysis 1.5 (Bruker, Karlsruhe, Germany). In case of present unwanted features in the image data, especially of scan line noise and bow, flattening operations (typically 0th and 1st order algorithms) have been performed. The presence of tilt artifacts in some images was reduced by applying plane fit operations to create a planar image profile.

### Sample origin

The author sincerely thanks for the donation of the biological samples investigated in this paper (*Caenorhabditis elegans* individuals were kindly provided by Eva Liebau (Institute of Animal Physiology, University of Münster, Germany), the knee cartilage sample was kindly donated by Thorsten Saenger (Institute of Pharmaceutical and Medicinal Chemistry, University of Münster, Germany), the human melanoma tissue was kindly provided by Hermann Schillers (Institute of Physiology II, University of Münster, Germany), *Ixodes hexagonus* individuals were kindly made available by Christina Strube (Institute of Parasitology, University of Veterinary Medicine Hannover, Germany) and mouse kidney samples were kindly donated by Martina Düfer (Institute of Pharmaceutical and Medicinal Chemistry, University of Münster, Germany). The samples not specifically mentioned were provided by the Institute for Pharmaceutical Biology and Phytochemistry (University of Münster, Germany).
